# Colocalization of expression transcripts with COVID-19 outcomes is rare across cell states, cell types and organs

**DOI:** 10.1007/s00439-023-02590-w

**Published:** 2023-08-28

**Authors:** Julian Daniel Sunday Willett, Tianyuan Lu, Tomoko Nakanishi, Satoshi Yoshiji, Guillaume Butler-Laporte, Sirui Zhou, Yossi Farjoun, J. Brent Richards

**Affiliations:** 1 Centre for Clinical Epidemiology, Department of Medicine, Lady Davis Institute, Jewish General Hospital, McGill University, 3755 Cote Ste Catherine, Pavillon H-413, Montréal, Québec H3T 1E2 Canada; 2 McGill University, Montreal, QC Canada; 3 Quantitative Life Sciences Program, McGill University, Montreal, QC Canada; 4 Department of Human Genetics, McGill University, Montreal, QC Canada; 5 Graduate School of Medicine, Kyoto-McGill International Collaborative Program in Genomic Medicine, Kyoto University, Kyoto, Japan; 6 Japan Society for the Promotion of Science, Tokyo, Japan; 7 Genome Centre, McGill University, Montreal, QC Canada; 8 Departments of Medicine, Human Genetics, Epidemiology and Biostatistics, McGill University, Montréal, QC Canada; 9 Department of Twin Research, King’s College London, London, UK; 10 Five Prime Sciences Inc, Montréal, Québec Canada

## Abstract

Identifying causal genes at GWAS loci can help pinpoint targets for therapeutic interventions. Expression studies can disentangle such loci but signals from expression quantitative trait loci (eQTLs) often fail to colocalize—which means that the genetic control of measured expression is not shared with the genetic control of disease risk. This may be because gene expression is measured in the wrong cell type, physiological state, or organ. We tested whether Mendelian randomization (MR) could identify genes at loci influencing COVID-19 outcomes and whether the colocalization of genetic control of expression and COVID-19 outcomes was influenced by cell type, cell stimulation, and organ. We conducted MR of *cis*-eQTLs from single cell (scRNA-seq) and bulk RNA sequencing. We then tested variables that could influence colocalization, including cell type, cell stimulation, RNA sequencing modality, organ, symptoms of COVID-19, and SARS-CoV-2 status among individuals with symptoms of COVID-19. The outcomes used to test colocalization were COVID-19 severity and susceptibility as assessed in the Host Genetics Initiative release 7. Most transcripts identified using MR did not colocalize when tested across cell types, cell state and in different organs. Most that did colocalize likely represented false positives due to linkage disequilibrium. In general, colocalization was highly variable and at times inconsistent for the same transcript across cell type, cell stimulation and organ. While we identified factors that influenced colocalization for select transcripts, identifying 33 that mediate COVID-19 outcomes, our study suggests that colocalization of expression with COVID-19 outcomes is partially due to noisy signals even after following quality control and sensitivity testing. These findings illustrate the present difficulty of linking expression transcripts to disease outcomes and the need for skepticism when observing eQTL MR results, even accounting for cell types, stimulation state and different organs.

**Supplementary Information:**

The online version contains supplementary material available at 10.1007/s00439-023-02590-w.

## Introduction

Severe COVID-19 is partially influenced by immune hyperstimulation (Kousathanas et al. 2022; Merad et al. 2022; Sharif-Zak et al. 2022; Tan et al. 2021). The immune response is mediated by different immune cell subtypes, including CD4 + T cells, acting at varying time points during infection across tissues (Ong et al. 2020; Tan et al. 2021). Understanding the dynamics of this process may pinpoint targets helpful for COVID-19 interventions, as previously shown (De Biasi et al. 2020; Degenhardt et al. 2022; Kundu et al. 2022; Mathew et al. 2020; Pairo-Castineira et al. 2022; Severe Covid et al. 2020; Wang et al. 2022; Zhou et al. 2021).

One way to investigate mechanisms influencing COVID-19 outcomes is to determine the underlying contributory genetic factors. GWAS has identified 87 loci associated with COVID-19 outcomes, but it is often unclear which gene(s) at such loci drive this association (Covid-19 Host Genetics Initiative 2021, 2022). Resolving a GWAS locus to its causal gene(s) is non-trivial (Forgetta et al. 2022). One way to identify causal genes at GWAS loci is to examine whether associated SNPs influence outcomes in an appropriate cell type. However, genetic determinants of gene expression (expression quantitative trait loci; eQTL) have often failed to “colocalize” with disease outcomes (Connally et al. 2022). Colocalization, in this context, means that gene expression and the disease outcome share a single common causal SNP (Connally et al. 2022). This lack of colocalization is concerning and not fully resolved. However, given the central importance of gene expression in disease incidence and progression, efforts are required to explain the paradox that the genetic determinants of gene expression often appear different than those of disease, even for known causal genes in known causal cell types or tissues (Connally et al. 2022). We sought to determine if this lack of colocalization could be explained when cell type, cell stimulation, method of sequencing and organ were taken into account.

One factor that may influence colocalization is the population of cells studied (Lamontagne et al. 2018). Gene expression is typically determined in bulk tissue, which provides a mixture of cells from the tissue. Such signal dilution, combined with complex factors such as cell–cell interactions, may explain why bulk tissue eQTLs often fail to colocalize with disease outcomes (Connally et al. 2022). Single-cell sequencing studies (scRNA-seq) assay gene expression in specific cell types and thus single-cell sequencing can provide a less heterogeneous assessment of gene expression. Comparing colocalization between bulk and single-cell sequencing, studying additional variables that influence the association, could resolve the contributory factors. This knowledge could help identify causal genes at GWAS loci and accelerate drug development by targeting pathways causal for disease. Targets supported by MR and colocalization evidence are more likely to anticipate clinical trial results, where the target of the medicine in the trial is a circulating protein and its causal influence upon disease is supported by both MR and colocalization. (Zheng et al. 2020).

Several studies have investigated the relationship between gene expression and COVID-19 outcomes using older releases of expression data or COVID-19 outcomes. Pairo-Castineira et al. found that increased *TYK2* and decreased *IFNAR2* expression in whole blood were associated with life-threatening COVID-19 (Pairo-Castineira et al. 2021). Schmiedel et al. found several genes whose expression in specific immune cell types and tissues, including resting and activated naive CD4 + cells, influenced and colocalized with genetic determinants of COVID-19 outcomes (Schmiedel et al. 2021). D’Antonio et al. found genes that colocalized with COVID-19 loci in whole blood, including *ABO* and *IFNAR2*, and identified the causal variants using fine-mapping (D'Antonio et al. 2021).

Recently, Soskic et al*.* profiled the changes in gene expression in CD4 + T cells following stimulation with anti-CD3/anti-CD28 human T activators (Soskic et al. 2022). We aimed to determine if cell and cell-state specific gene expression could identify novel determinants of COVID-19 outcomes, suggesting which cells are responsible for COVID-19 mortality risk and when. Further, we aimed to determine if such cellular specificity may clarify colocalization of expression and GWAS data. Repeating this analysis in other cell types, bulk whole-blood in individuals with symptoms of COVID-19, with and without a recent PCR-confirmed infection, and bulk tissues in individuals assessed prior to the pandemic lacking any symptoms would identify differences in colocalization due to sequencing modality and different clinical states. The results would afford insights into whether the genetic control of gene expression and disease risk is clarified when resolving to single cells, specific cellular states and clinical characteristics of patients sampled.

**Fig. 1:**
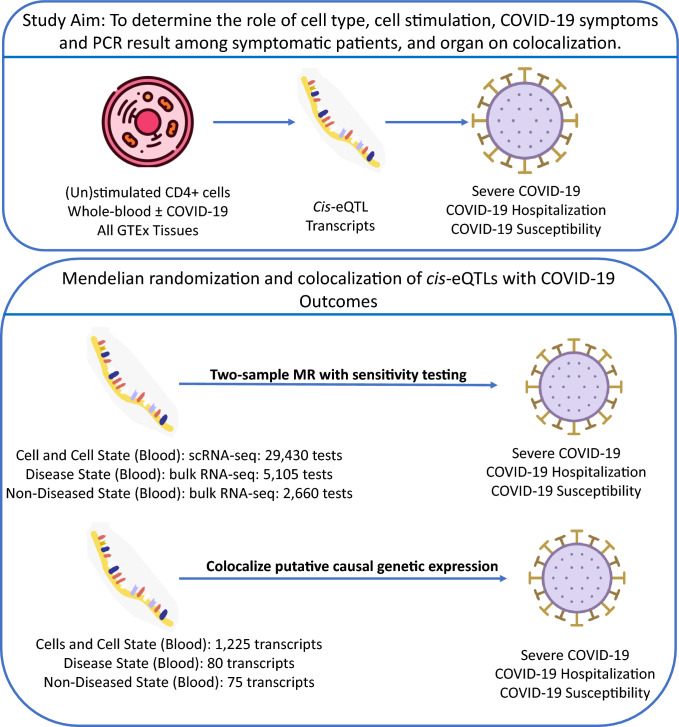
Study overview

To answer these questions, we undertook a four-stage study design (Fig. 1). First, we conducted Mendelian randomization (MR) of *cis*-eQTLs obtained from single-cell RNA-sequencing (scRNA-seq) data from CD4 + T-cell subtypes at varying times after stimulation with gene expression as exposures and COVID-19 severity as an outcome. Second, we tested these MR-identified genes for colocalization with COVID-19 outcomes across all time points following stimulation of CD4 + T cells. Third, we compared these colocalization results to those from bulk whole-blood RNA sequencing obtained from individuals with COVID-19 symptoms, who were either SARS-CoV-2 PCR positive or negative. Fourth, we compared the colocalization results to bulk unstimulated whole blood and 47 other tissues obtained from individuals assessed in GTEx v.8, whose tissues were apparently undiseased at time of sampling.

These findings identified 33 genes whose expression may influence COVID-19 outcomes. Colocalization was highly variable and not consistent across the factors that may influence it. These results underline the complexity of factors that influence the colocalization of genetic expression with disease.

## Results

### Cohort demographics

All datasets used in this study included individuals solely of European ancestry to reduce potential confounding by population stratification. ScRNA-seq data for CD4 + T cells in whole blood was obtained from Soskic et al*.*, who isolated cells from 119 healthy, British-ancestry individuals, with a mean age of 47 years, where 44% were females (Soskic et al. 2022). Bulk whole-blood RNA sequencing of individuals with symptoms of COVID-19, who were SARS-CoV-2 positive or negative, was obtained from BQC19, a Quebec cohort of individuals recruited from hospitals presenting with COVID-19 symptoms. BQC19 RNA-sequencing data comprised 112 individuals with symptoms of COVID-19 and SARS-CoV-2-positive PCR tests and 166 individuals who also had symptoms of COVID-19 but a SARS-CoV-2 negative PCR test. The mean age of BQC19 participants was 54 years and 53% were females. The GTEx Consortium cohort comprised 838 post-mortem donors, including 715 individuals of European ancestry, of which 33.5% were female (GTEx Consortium 2020).

### MR and sensitivity testing

**Fig. 2:**
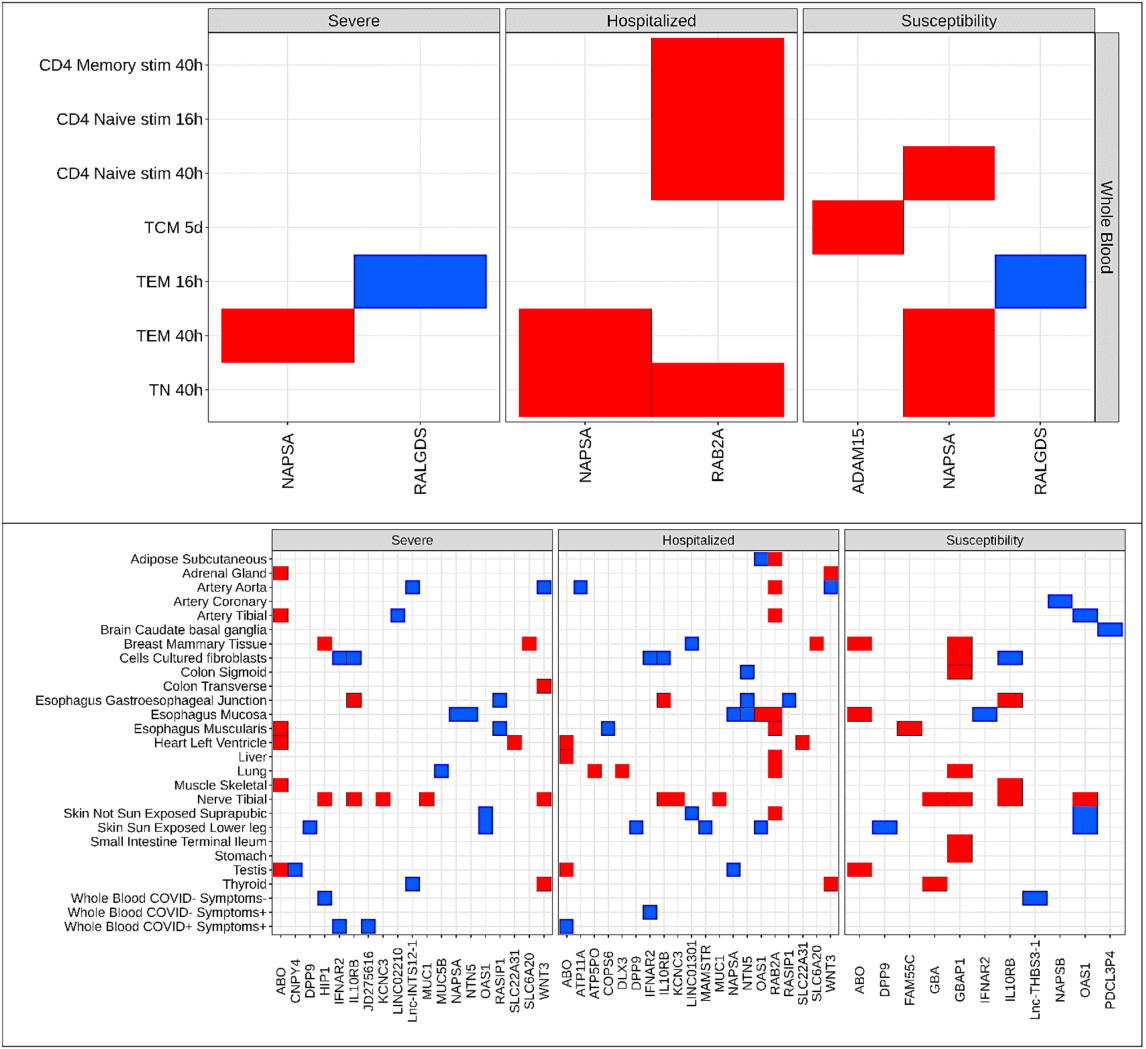
A total of 33 genes that colocalized across body tissue, evaluated at the **a** single-cell and **b** bulk tissue level, were estimated to have their expression increase (red) and decrease (blue) risk of COVID-19 outcomes. In bulk tissue, COVID + referred to individuals who had tested positive for COVID-19 with COVID- for those who tested negative and Symptoms + refers to individuals who presented with symptoms of COVID-19 with Symptoms- referring to those who did not have symptoms of COVID-19. COVID- Symptom- refers to GTEx whole blood. Note that some transcripts show increased expression in some tissues to be associated with COVID-19 outcomes, whereas other tissues show decreased expression to be associated with the same transcript for the same outcome. *TCM* T central memory cell. *TEM* T effector memory cell. *TN* T naïve cell

To identify genes influencing COVID-19 outcomes, we used either a Wald ratio or inverse-variance weighted MR analysis at 81 non-MHC COVID-19 associated loci (Supplemental Table 1) across three COVID-19 outcomes (severe disease, hospitalized disease, and susceptibility to disease) with sensitivity analyses for each test, detailed in Methods. We identified 33 genes whose expression was shown by MR to influence COVID-19 severity and susceptibility (Fig. 2, Table 1, Supplemental Table 2).

**Table 1:** Putatively causal associations of gene expression with COVID-19 outcomes

Gene	Putatively causal cell/tissue	Existing evidence
*ABO*	Adrenal gland (Severe)—Hazardous Artery tibial (Severe)—Hazardous Breast Mammary Tissue (Susceptible)—Hazardous Esophagus mucosa (Susceptible)—Hazardous Esophagus muscularis (Severe)—Hazardous Heart Left Ventricle (Severe and Hospitalized)—Hazardous Liver (Hospitalized)—Hazardous Muscle skeletal (Severe)—Hazardous Testis (All)—Hazardous Whole blood COVID + Symptoms + (Hospitalized)—**Protective**	MR in non-GTEx and GTEx eQTL and pQTL datasets for lung and whole blood (Baranova et al. 2022a; Hernandez Cordero et al. 2021)
*ATP5PO*	Lung (Hospitalized)—Hazardous	Associational (Li et al. 2022)
*GBAP1*	Breast Mammary Tissue (Susceptible)—Hazardous Cultured Fibroblasts (Susceptible)—Hazardous Colon Sigmoid (Susceptible)—Hazardous Lung (Susceptible)—Hazardous Nerve Tibial (Susceptible)—Hazardous Small Intestine Terminal Ileum (Susceptible)—Hazardous Stomach (Susceptible)—Hazardous	MR with colocalization for splice isoform (Nakanishi et al. 2022)
*HIP1*	Breast mammary tissue (Severe)—Hazardous Nerve tibial (Severe)—Hazardous Whole blood COVID-symptoms-(Severe)—**Protective**	None
*IFNAR2*	Cells cultured fibroblasts (Severe and hospitalized)—Protective Esophagus mucosa (Susceptible)—Protective Whole blood COVID- Symptoms + (Hospitalized)—Protective Whole blood COVID + Symptoms + (Severe)—Protective	MR with colocalization for GTEx v.7 eQTLs and pQTLs for whole-blood and lung (Baranova et al. 2021; Fricke-Galindo et al. 2022; Krishnamoorthy et al. 2023; Liu et al. 2021; Pairo-Castineira et al. 2021)
*IL10RB*	Cells Cultured fibroblasts (All)—**Protective** Esophagus Gastroesophageal junction (All)—Hazardous Skeletal muscle (Susceptible)—Hazardous Nerve tibial (All)—Hazardous	MR with colocalization for eQTLs and pQTLs, did not study excitatory neurons (Gaziano et al. 2021)
*RAB2A*	Adipose subcutaneous (Hospitalized)—Hazardous Artery aorta (Hospitalized)—Hazardous Artery tibial (Hospitalized)—Hazardous CD4 Memory Stim 40 h (Hospitalized)—Hazardous CD4 Naïve Stim 16 h (Hospitalized)—Hazardous CD4 Naïve Stim 40 h (Hospitalized)—Hazardous Esophagus mucosa (Hospitalized)—Hazardous Esophagus muscularis (Hospitalized)—Hazardous Liver (Hospitalized)—Hazardous Lung (Hospitalized)—Hazardous Skin not sun exposed (Hospitalized)—Hazardous T naïve Stim 40 h (Hospitalized)—Hazardous	MR and colocalization for pQTLs, but not for eQTLs (Pietzner et al. 2022)
*RALGDS*	T effect memory Stim 16 h (Severe and Susceptible)—Protective	None
*WNT3*	Adrenal Gland (Hospitalized)—Hazardous Artery Aorta (Severe and Hospitalized)—**Protective** Colon Transverse (Severe) – Hazardous Nerve Tibial (Severe) – Hazardous Thyroid (Severe and Hospitalized) – Hazardous	Fine mapped (Wu et al. 2021)

While some genes had opposite effects on risk for a minority of tissues (bolded), the direction of effect of most was consistent when observed in multiple tissues

*Single-cell results*. Of the 29,430 combinations of CD4 + cell:stimulation-state:gene:outcome for the whole-blood single-cell eQTLs, 1,225 transcripts (4.2%) had a multiple-testing, Benjamini–Hochberg adjusted p-value ≤ 0.05 in the MR analyses. We limited results to only those arising from *cis*-eQTLs to reduce potential bias from horizontal pleiotropy. *Cis*-eQTLs were defined as genetic variants associated with transcript level within ± 500 kb of the transcriptional start site. We also only retained *cis*-eQTLs that were within ± 500 kb of the lead SNP associated with a COVID-19 outcomes (Supplemental Table 3). Only 13 of these 1,225 *cis*-eQTLs passed colocalization sensitivity testing, defined as having a probability of the gene’s expression and the COVID-19 outcome sharing a single causal variant (PP_H4_) greater than 0.80 (Fig. 1, 2). These data demonstrate that only a small proportion of *cis*-single-cell eQTLs identified via MR colocalized with COVID-19 outcomes, suggesting that such MR findings do not consistently reflect a common causal genetic signal shared between the transcript and COVID-19 outcomes.

### Bulk results

Next, we assessed *cis*-eQTLs from the bulk whole-blood RNA sequencing in the BQC19 cohort, which comprised individuals with symptoms of COVID-19 who had either a positive, or negative PCR test for SARS-CoV-2. Of the 5105 combinations of study-group:gene:outcome for the BQC19 *cis*-eQTLs, 80 transcripts (1.6%) were identified by MR to have effects on COVID-19 outcomes (Supplemental Table 4). Only 4 of these 80 transcripts passed colocalization sensitivity testing (Figs. 1, 2). We next assessed whether we would observe similar results if we used whole blood and other organ bulk RNA sequencing from GTEx v.8 (GTEx Consortium 2020) as the source of the cis-eQTLs, that included individuals without symptoms or seropositivity for COVID-19. In GTEx bulk whole-blood, we found that of the 2,660 combinations of gene:outcome for the GTEx *cis*-eQTLs, 75 (2.8%) were identified by MR testing to have effects upon COVID-19 outcomes, with only two colocalizing (Figs. 1, 2). In all 48 GTEx tissues, we found that of the 125,088 combinations of tissue:gene:outcome tested, 3190 (2.6%) were estimated to influence COVID-19 outcomes by MR but only 98 of these colocalized (Fig. 2).

Thus, taken together, across the three different sources of gene expression data (scRNA-seq whole blood CD4 + T cells, bulk whole blood RNA sequencing in patients with symptoms of COVID-19, and bulk RNA sequencing in individuals whose tissues were apparently health across 48 tissues, including whole blood), we observed 33 unique putatively causal transcripts across 115 specific states that colocalized, which represent 2.3% of those transcripts that survived MR testing and multiple testing thresholds. These findings suggest that most transcripts identified to be associated with COVID-19 outcomes via MR fail to colocalize even across single cell and bulk sequencing, as well as different cellular states and patient states.

### MR implicated several cis-eQTLs that increase or decrease the risk for COVID-19 outcomes with few overlapping transcripts between scRNA-seq and bulk RNA-sequencing results

Across outcomes, all cell types from the scRNA-seq whole-blood data had at least one *cis*-eQTL estimated to be causal for a COVID-19 outcome via MR, except T effector memory re-expressing CD45RA and T regulatory cells (Fig. 2). Of the four genes estimated to have colocalized causal effects from the whole-blood scRNA-seq MR experiments, *RALGDS* expression was the only *cis*-eQTL that decreased the risk of COVID-19 outcomes. Specifically, *RALGDS* expression reduced the risk of severe disease (OR = 0.78, 95% CI 0.71–0.87; adjusted *p = *1.1 × 10^–4^) and susceptibility to disease (OR = 0.88, 95% CI 0.86–0.90; adjusted *p = *7.4 × 10^–18^), when expressed in solely T effector memory cells 16 h after stimulation (Fig. 2). *RALGDS’* influence on hospitalized COVID-19 was not clearly different from the null (OR = 0.87, 95% CI 0.79–0.96; adjusted *p = *0.08) but passed MR sensitivity testing and colocalized. *NAPSA* expression in T naive cells 40 h after stimulation increased risk of hospitalized (OR = 1.17, 95% CI 1.08–1.28; adjusted *p = *5.0 × 10^–3^) and susceptibility to (OR = 1.08, 95% CI 1.05–1.11; adjusted *p = *1.7 × 10^–5^) COVID-19 (Fig. 2). *NAPSA’*s influence on severe COVID-19 was not different from the null (OR = 1.20, 95% CI 1.06–1.37; adjusted *p = *0.07) but passed MR sensitivity testing and colocalized. *NAPSA* also increased risk for every outcome in T effector memory cells 40 h after stimulation (OR = 1.18, 95% CI 1.10–1.27; adjusted *p = *4.0 × 10^–4^ for severe, OR = 1.15, 95% CI 1.10–1.20; adjusted *p = *5.9 × 10^–9^ for hospitalized, OR = 1.04, 95% CI 1.03–1.06; adjusted *p = *1.2 × 10^–4^ for susceptibility) (Fig. 2).

Interestingly, none of the colocalizing transcripts from scRNA-seq overlapped with colocalizing bulk RNA-sequencing *cis*-eQTLs from BQC19 or GTEx in matching tissues. Generally, signals in bulk sequencing were harder to separate from noise, compared to single-cell results. Increased *IFNAR2* expression was protective in individuals with symptoms of COVID-19 without PCR-confirmed SARS-CoV-2 against severe COVID-19, or individuals who were negative for or had perhaps not yet tested positive for COVID-19 (OR = 0.75, 95% CI 0.66–0.86; adjusted *p = *3.0 × 10^–3^) with insufficient evidence to suggest protection for individuals who did test positive (adjusted *p = *0.04) where it failed weighted mode MR sensitivity testing but colocalized. In contrast, *IFNAR2* was protective for only individuals who tested positive for SARS-CoV-2 against hospitalized COVID-19 (OR = 0.86, 95% CI 0.80–0.94; adjusted *p = *0.03) with insufficient evidence to suggest protection for BQC19 SARS-CoV-2-negative individuals (adjusted *p = *8.1 × 10^–3^) where it failed MR Egger intercept sensitivity testing (*p = *0.02) while colocalizing (Fig. 2). This was perhaps a false negative. There were several other genes that colocalized for only a subset of outcomes in other tissues, although some consistently colocalized in the same tissue across outcomes, such as ABO in the testis and DPP9 in sun-exposed skin (Table 1, Supplemental Table 2).

### Colocalization of specific MR-identified cis-eQTLs depended on cell stimulation

**Fig. 3:**
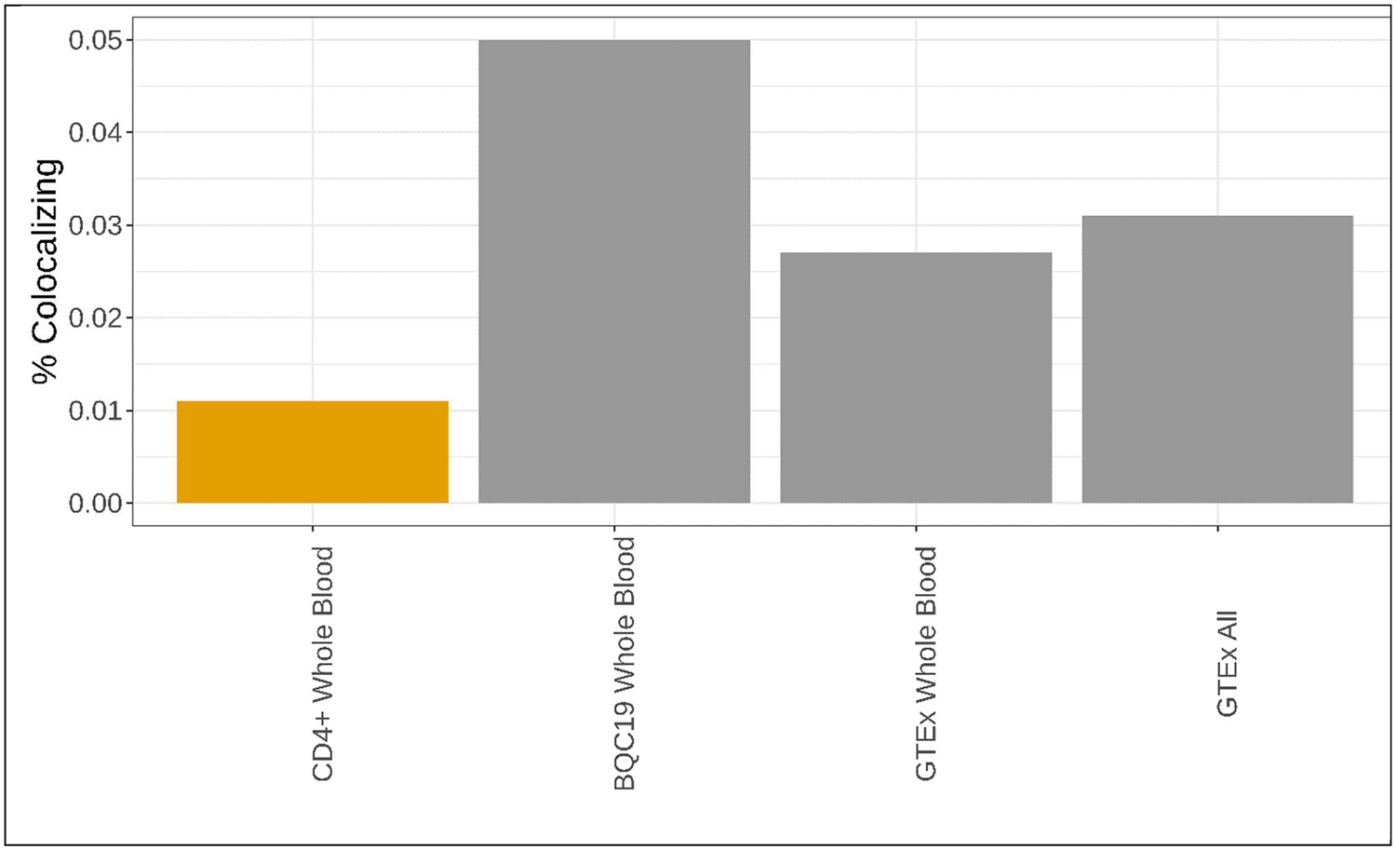
The proportion of estimated causal variants that colocalized and passed sensitivity testing. Orange bars refer to single-cell data, gray bulk sequencing

**Fig. 4:**
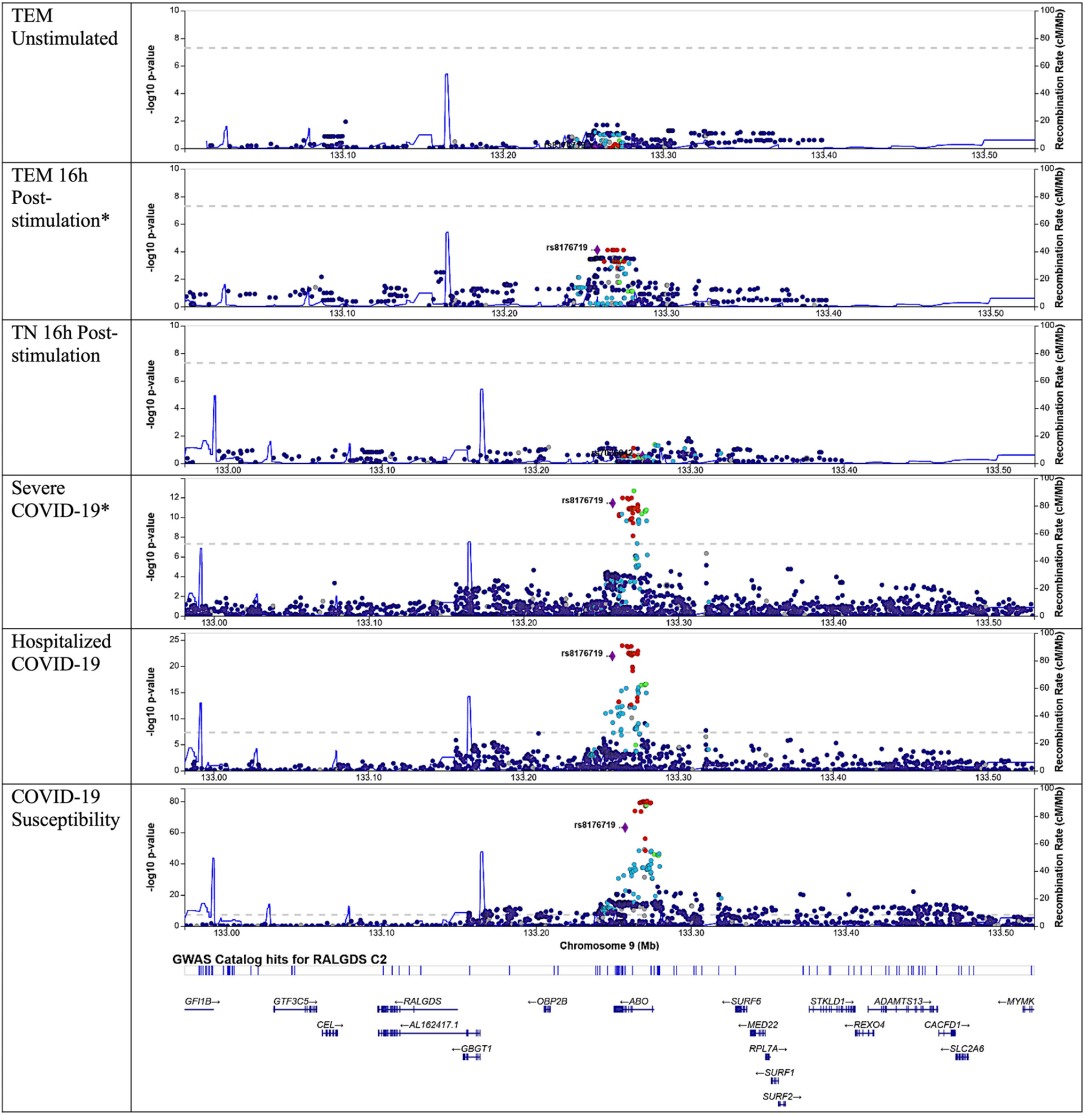
Locuszoom plots for *RALGDS* in the setting of variable cell stimulation and cell type, with selected variant of rs8176719. *RALGDS* was estimated causal with colocalization for only T effectory memory (TEM) cells 16 h post-stimulation, highlighting the role of cell stimulation on colocalization. A *indicates a dataset that colocalized. rs8176719 was not detected in T naïve cells 16 h post-stimulation, so rs7036642 was highlighted, which is in LD with rs8176719

To investigate the variables that influenced colocalization, we conducted colocalization on each gene that was estimated causal in MR. Most MR-identified transcripts did not colocalize (Fig. 1, Supplemental Tables 6–8). The proportion of estimated causal transcripts that colocalized and passed single causal variant sensitivity testing was 1.1% for whole blood single-cell eQTLs, 5.0% for BQC19, 2.7% for GTEx whole blood, and 3.1% for all organs in GTEx (Fig. 3). Colocalization of 2/4 single-cell eQTLs was specific to cell type (Fig. 2) with 3/3 from stimulated cells specific to cell state with colocalization for *RALGDS*, for example, specific to T effector memory cells 16 h post-stimulation for severe and susceptibility to COVID-19 (Fig. 4). While RALGDS was mapped, its contributory *cis*-eQTLs were mapped around ABO, which has been suggested to mediate COVID-19 pathogenesis via glycosylation of downstream targets and colocalized in others’ studies, underscoring the complexity of mapping eQTLs (Hernandez Cordero et al. 2021; Wang et al. 2021).

**Fig. 5:**
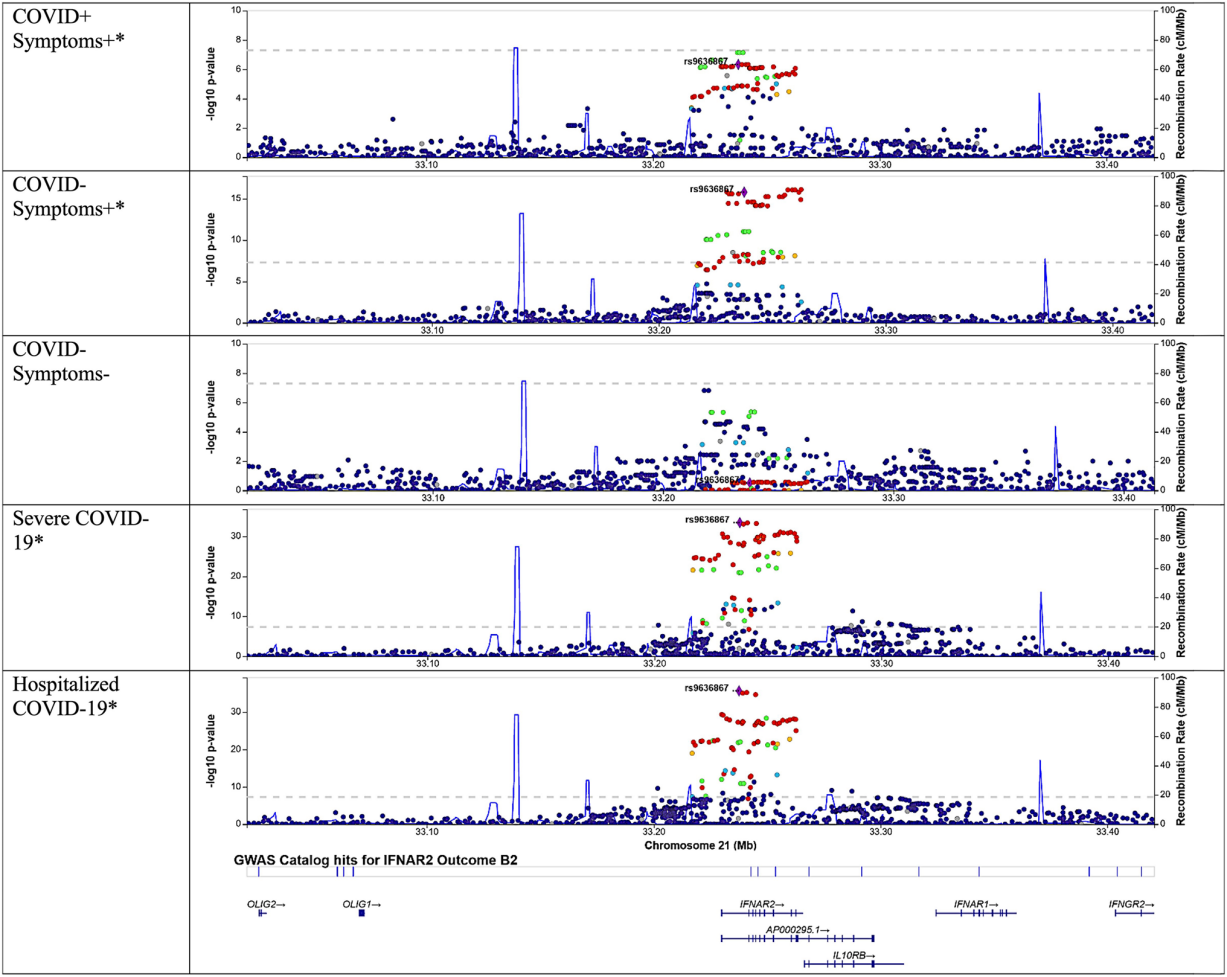
Locuszoom plots for *IFNAR2* in the setting of variable presence of COVID-19 symptoms and SARS-CoV-2 positivity, with selected variant of rs9636867. *IFNAR2* was estimated causal and colocalized for severe COVID-19 only for individuals with a positive SARS-CoV-2 titer and symptoms (COVID + Symptom +) and for hospitalized COVID-19 only for individuals with a negative SARS-CoV-2 titer and symptoms (COVID- Symptom +), underscoring the role of sample size and linkage disequilibrium on signals. A *indicates a dataset that colocalized

**Fig. 6:**
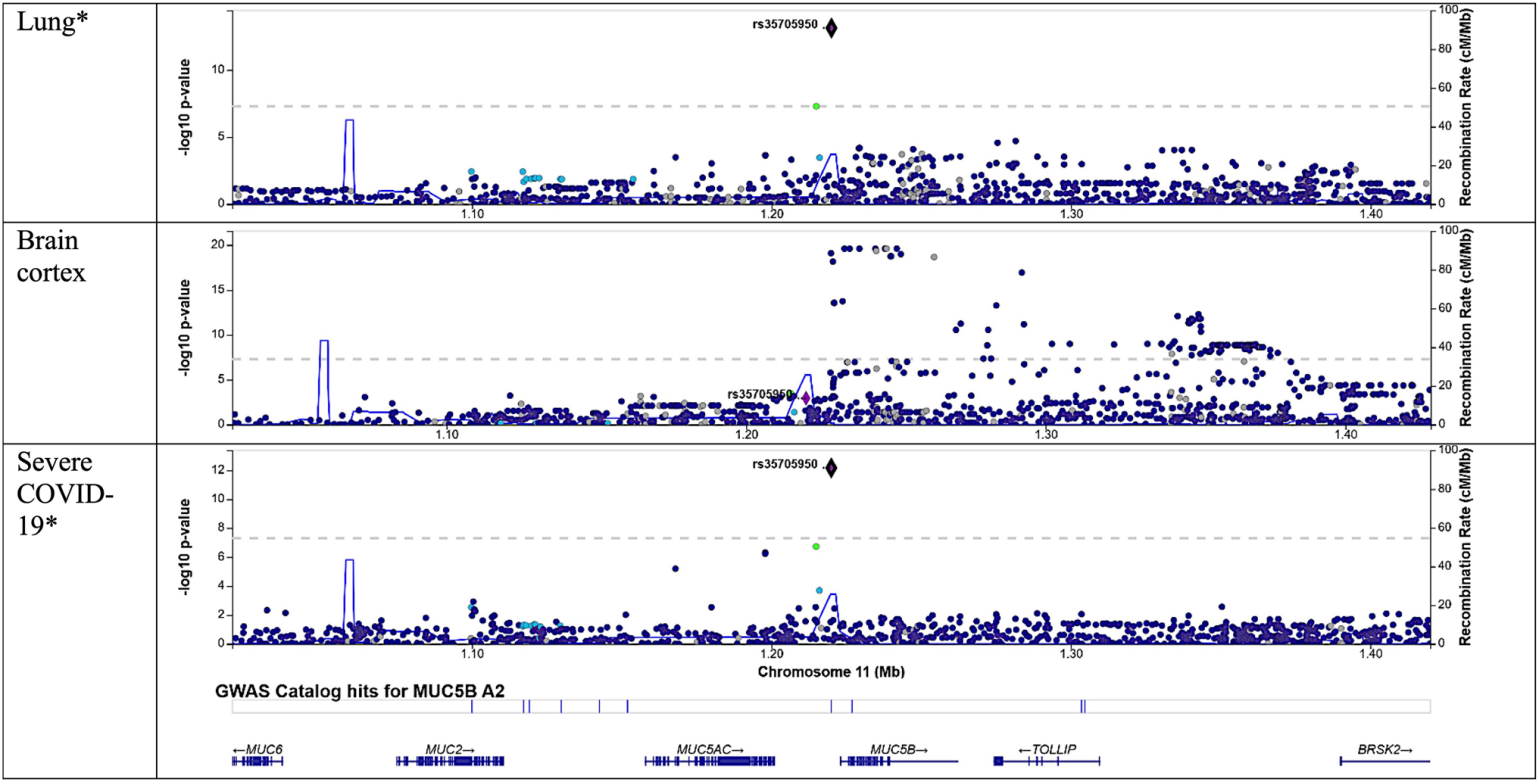
Locuszoom plots for *MUC5B* in the setting of variable organ in GTEx v8, with selected variant of rs35705950. *MUC5B* was estimated causal and colocalized only in lung for severe COVID-19. There were no *cis*-eQTLs for whole blood

Colocalization of transcripts occurred at varying times following stimulation (Fig. 2). Colocalization did not appear to depend on SARS-CoV-2 PCR result in BQC19 among individuals with symptoms of COVID-19, as seen with *IFNAR2* (Figs. 2, 5). Colocalization of select transcripts appeared organ specific, as with *MUC5B* (Fig. 6), although there was more noise in bulk sequencing data, as was observed for *ABO.* Specifically, *ABO* colocalized in some organs for only a single outcome and *IL10RB* tested in GTEx had an opposite direction of effect in cultured fibroblasts than tibial nerve (Fig. 2).

**Fig. 7:**
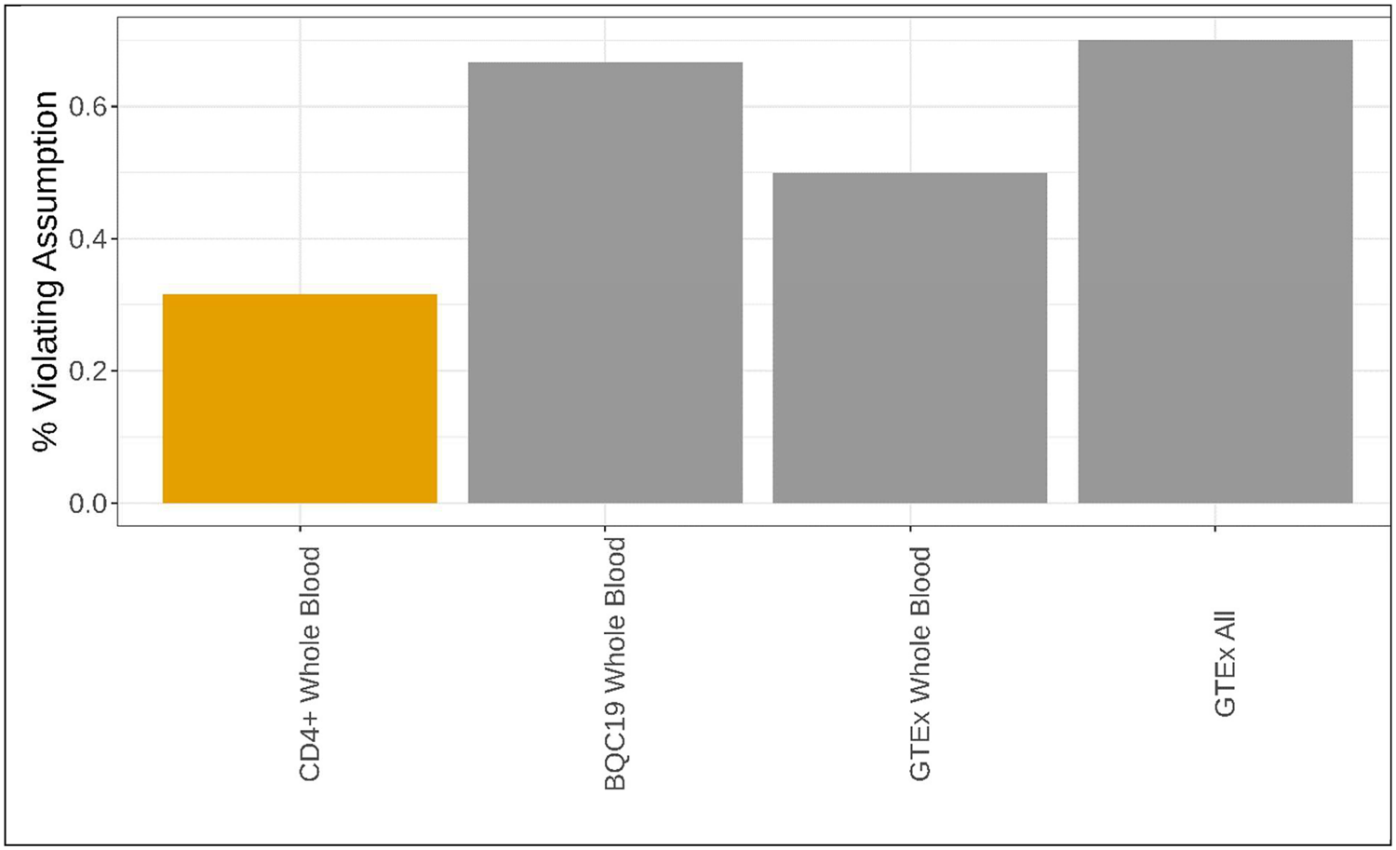
Percentage of colocalizing results (PP_H4_ ≥ 0.80) that had evidence of violating the single causal variant assumption. Orange bars refer to single-cell data, gray bulk sequencing

While many transcripts did not colocalize, many transcripts that did have evidence of the single causal variant assumption being violated, with multiple peaks present within or close to the cognate 1 Mb window, suggesting bias due to linkage disequilibrium (Fig. 7). Of 19 colocalizing transcripts in single-cell CD4 + T cells, 6 had evidence of violating this assumption. Of nine colocalizing transcripts in bulk whole blood from patients with symptoms of COVID-19 in BQC19, we observed six that violated this assumption. Of four from bulk whole blood in GTEx, two violators. Of 378 from all tissues in GTEx, 265 transcripts violated this assumption. These results underpin the limitations of present study sample sizes and existing methods designed to clarify causal colocalizing expression signals.

## Discussion

In this study, we attempted to identify factors that influence colocalization of *cis*-eQTL MR findings for COVID-19 outcomes. Most MR-identified single-cell eQTLs and bulk eQTLs did not colocalize, suggesting that linkage disequilibrium and limited study sizes yielding fewer converging signals may strongly impact the validity of eQTL MR studies that assess colocalization, and necessitate more stringent quality control. Previous research has demonstrated a lack of colocalization of expression findings and suggested that this may be resolved by single-cell eQTL analyses, assessing stimulation state, or clearly defining the state of the individual when blood samples were drawn. Here, we show that colocalization of these signals is highly variable, and it is not fully explained by changing cell type, cell stimulation, symptoms of COVID-19, or organ. Taken together, these findings suggest that colocalization of eQTLs with disease outcomes is difficult using current technologies, reference panels and statistical methods, and with present study sample sizes that in single-cell studies typically is limited to 100 individuals.

While we overall found colocalization to be limited, our results were reassuring in that we observed some trends and results consistent with past studies and hypotheses (Fig. 2) (Connally et al. 2022; Soskic et al. 2022). For putatively causal transcripts from CD4 + T cells in whole blood, colocalization was influenced by cell stimulation, or cell state (Fig. 2a), like other groups’ observations when conducting colocalization without MR (Soskic et al. 2022). On comparing putatively causal transcripts between single-cell and bulk results, with the latter evaluating transcripts in several different cells in a matching tissue, we found no overlap between the modalities in the same tissue, that could suggest that sequencing modality plays a role (Fig. 2). Genes that colocalized in CD4 + T cells did not colocalize in spleen that is enriched with T cells (Fig. 2). This could underscore the impact of sequencing multiple cell types in bulk sequencing, suggesting that results must be contextualized against sequencing modality and sample cellular heterogeneity. We found organ to play a moderate role in colocalization when comparing putatively causal genes in GTEx (Fig. 2b), supporting others’ findings with non-COVID-19 outcomes (Rocheleau et al. 2022). The data on organs’ roles were perhaps noisy. While we found outcome to have a limited role in influencing colocalization for select transcripts, different from others (Rocheleau et al. 2022; Soskic et al. 2022), this could be due to the greater similarity in our outcomes.

This paper has limitations. The scRNA-seq data came from cells stimulated by a standard T-cell stimulator rather than SARS-CoV-2. Such stimulation has been employed by several existing works studying immunity in COVID-19 (De Biasi et al. 2020; Kundu et al. 2022; Mathew et al. 2020) and may have some relevance to the stimulation endured when T cells encounter SARS-CoV-2. We only investigated CD4 + T cells in whole blood (Fig. 2). There are other cell types present in greater proportions, which could explain the minimal overlap in colocalization between our single-cell and bulk sequencing results. We could only locate whole blood *cis*-sceQTLs from an admixed study that did not release ancestry-stratified results (Randolph et al. 2021). Ancestry-stratified results are important for Mendelian randomization and colocalization to limit bias from indirect pleiotropic mechanisms, including linkage disequilibrium that varies by ancestral group. Our GTEx results show that many tissues could contribute to COVID-19 outcomes and pathogenesis (Fig. 2). Single-cell analysis of all tissues, particularly lung, could help understand the multi-system basis of post-COVID-19 syndrome (Mehandru and Merad 2022). We were unable to access single-cell lung data for this work (Lamontagne et al. 2018). Our outcomes were limited to COVID-19 severity and susceptibility, when expression could also influence complications of COVID-19, such as post-COVID-19 syndrome and schizophrenia (Baranova et al. 2022b; Mehandru and Merad 2022). We have developed two-step MR methods that link gene expression to COVID-19 outcomes, and then to these complications (Yoshiji et al. 2023), which has already been used to validate BMI’s role on COVID-19 outcomes as discovered by others (Baranova et al. 2023).

Our study was limited to individuals of European ancestry. While the HGI has found loci with genome-significant variants in other ancestries, none of the loci from Admixed American, African, East Asian, or South Asian ancestry for any outcome overlapped with loci found to influence COVID-19 outcomes in this study. Given fewer loci in non-European datasets, this could be due to sample size and underscores the importance of multi-ancestry analyses. However, even within European ancestry individuals, subtle differences in LD patterns can influence colocalization, which we observed (Figs. 4, 5, 6) (Kanai et al. 2021, 2022). Such differences may have impacted the lack of colocalization observed here. Sample size was limited for the eQTL datasets that we employed, which increases the risk of biased results, which we possibly observed where some genes colocalized for only a single outcome or did not colocalize in one outcome (Fig. 2). We adjusted for this by using methods well established in the literature, including several stringent and conservative sensitivity tests and means of quality control for MR and colocalization. While using a colocalization window around a lead variant of ± 500 kb is more likely to limit bias from pleiotropy, it increases the risk of missed signals. Data must still be carefully appraised for possible false positives, as may be the case with *IL10RB* being estimated protective in cultured fibroblasts for all outcomes but increasing the risk of COVID-19 outcomes in the tibial nerve for all outcomes (Fig. 2).

While we found symptoms of COVID-19 to influence genomic colocalization for select transcripts and states, as shown by *IFNAR2* between severe and hospitalized COVID-19, this trend could have been influenced by a batch effect. SARS-CoV-2 status’ effect on colocalization among individuals in BQC19 could be biased as negative results could represent false negatives and individuals with COVID-19 symptoms could have had a different viral illness. We mitigated this by deriving eQTLs using the same pipeline used by GTEx and limiting the conclusions we made given this context (GTEx Consortium 2020). Generally, we investigated variables in multiple datasets, given that a variable’s demonstrated role in multiple cohorts supports making a generalization.

## Conclusions

While existing hypotheses suggest that colocalization of transcripts depends on multiple conditions, we found that *cis*-eQTLs identified by MR for COVID-19 outcomes rarely colocalized, even when assessing different cell types, cell states, symptoms of COVID-19 and organs. Taken together, these findings suggest that even after accounting for variables, there was little evidence of colocalization for most genes whose influence on COVID-19 outcomes was identified through MR.

## Methods

### Datasets

We examined *cis*-eQTLs in three datasets to implicate their influence on COVID-19 outcomes and investigate how experimental and physiological conditions impact colocalization (Fig. 1). scRNA-seq *cis*-eQTLs from Soskic et al*.* were used to analyze how cell type, cell stimulation, and cell microenvironment affected colocalization (Soskic et al. 2022). Bulk RNA-seq *cis*-eQTLs from Biobanque Quebecoise de la COVID-19 (BQC19) were used to investigate how disease state impacted identified *cis*-eQTLs and colocalization in individuals with symptoms of COVID-19, with and without PCR-confirmed SARS-CoV-2 infection. Bulk RNA-seq *cis*-eQTLs from GTEx whole blood were used to compare BQC19 data with data from individuals without symptoms of COVID-19 with all other organs used to determine the role of organ (GTEx Consortium 2020).

### Whole-blood single-cell eQTLs

The summary statistics for immune cell expression *cis*-eQTLs before and after stimulation with anti-CD3/anti-CD28 human T-Activators were obtained from Soskic et al. (Soskic et al. 2022). The study consisted of 119 individuals of British ancestry with peripheral blood mononuclear cells (655,349 CD4 + T cells) sequenced using scRNA-seq (Soskic et al. 2022). We used the summary statistics for cells at all available time points (unstimulated, 16 h post-stimulation corresponding to before cell division, 40 h post-stimulation corresponding to after cell division, 5 days post-stimulation corresponding to gaining effector function) for cell types present before stimulation and present for at least one time point after stimulation (Soskic et al. 2022). The unstimulated time point acted as a control for stimulation, sequenced 16 h after culturing without any anti-CD3/anti-CD28 human T-Activators (Soskic et al. 2022). We investigated CD4 + antigen-I and CD4 + memory cell classifications before Leiden-algorithm clustering, implemented by Soskic et al., and T I, T central memory, T effector memory, CD45RA re-expressing T effector memory, and thymus-derived regulatory T cells after clustering (Soskic et al. 2022). Full details describing RNA sequencing, separation of cell types and stimulation are available in Soskic et al. (Soskic et al. 2022).

### Bulk whole blood eQTLs from subjects with symptoms of COVID-19, with and without SARS-CoV-2-positive PCR results

BQC19 (https://en.quebeccovidbiobank.ca) is a prospective cohort enrolling participants with PCR-proven SARS-CoV-2 infection and PCR-proven SARS-CoV-2 negative individuals who presented to the hospital with signs or symptoms consistent with COVID-19. Participants were recruited from eight academic hospitals in the province of Quebec, Canada. A total of 4704 participants underwent PCR testing with confirmed positive or negative results between January 25^th^, 2020 and March 20^th^, 2022. A total of 379 participants had RNA extracted and sequenced with eQTLs called using the GTEx pipeline, available from the Broad Institute (https://github.com/broadinstitute/gtex-pipeline/tree/master/qtl). We used data solely for those of non-Finnish European ancestry, which included 112 SARS-CoV-2-positive and 166 SARS-CoV-2-negative samples.

### GTEx release 8 data

We obtained *cis*-eQTL summary statistics for GTEx release 8 for whole-blood and all other organs, restricted to individuals of European ancestry, from: https://www.gtexportal.org/home/ (GTEx Consortium 2020).

### COVID-19 outcome data

European-ancestry-specific summary statistics for COVID-19 outcomes were obtained from the COVID-19 Host Genetics Initiative (HGI) release 7 (https://www.covid19hg.org/), which did not include individuals from 23AndMe (Covid-19 Host Genetics Initiative 2021, 2022). The COVID-19 outcomes included severe COVID-19 (13,769 cases and 1,072,442 controls), COVID-19 hospitalization (32,519 cases and 2,062,805 controls), and susceptibility to COVID-19 (122,616 cases and 2,475,240 controls). Severe COVID-19 was defined as COVID-19 requiring respiratory support or resulting in death. COVID-19 with hospitalization was defined as an infection requiring hospitalization or death. COVID-19 susceptibility was defined as infection determined by self-report on a questionnaire, electronic medical record diagnosis, or laboratory testing (serology tests or nucleic acid amplification). Controls were all individuals who did not meet an outcome’s definition.

### MR of cis-eQTLs with COVID-19 outcomes

We used MR to estimate the causal relationship between exposures (which here are RNA transcript levels) and COVID-19 outcomes to determine how cell type, cell stimulation, time after stimulation, symptoms of COVID-19, PCR result for SARS-CoV-2 among individuals with symptoms of COVID-19, and organ influenced the relationship between gene expression and COVID-19 outcomes. MR studies use SNPs strongly associated with an exposure as instrumental variables to estimate the effect of an exposure on an outcome. Such studies reduce potential confounding effects, because genetic variants are essentially randomized at conception. They also prevent bias due to reverse causation (wherein the outcome influences the exposure) since the assignment of genetic variants always precedes disease onset (Smith and Ebrahim 2003). The main assumptions of MR are that the variants under study are strongly associated with the risk factor of interest, confounders of the exposure-outcome relationship are not associated with the variants, and the variants only affect the outcome through the risk factor (Davies et al. 2018; Skrivankova et al. 2021). The most problematic of these assumptions is the last, as it is difficult to confidently understand if the SNPs affect the outcome independent of the exposure (i.e. a lack of horizontal pleiotropy). To partially mitigate against such potential bias, we have used only *cis*-eQTLs, which are more likely to act directly through the transcription or translation of the proximal gene, rather than through horizontally pleiotropic pathways. There was overlap of individuals from the *cis*-eQTL studies with individuals in the HGI data.

To undertake MR analyses, we used the TwoSampleMR (v0.5.6) package (https://mrcieu.github.io/TwoSampleMR/). First, we identified all genome-significant (p ≤ 5 × 10^–8^) loci from HGI COVID-19 summary statistics. We isolated all variants 500 kb upstream and downstream of the lead HGI variant for each locus for each exposure and outcome. Second, we excluded the MHC locus (chr6: 28,510,120 – 33,480,577; GRCh38) to reduce potential confounding by linkage disequilibrium structure. Third, the exposure *cis*-eQTLs, ± 500 kb from their transcriptional start sites, for each cell type, cell stimulation state, cell stimulation time, SARS-CoV-2 status, presence of COVID-19 symptoms, and gene were filtered for variants in LD using the package’s clumping function with default settings. Fourth, the exposure data were harmonized with outcome data using default settings. Fifth, we conducted MR with sensitivity tests. For loci with only one remaining *cis*-eQTL following harmonization, we applied Wald Ratio-based MR. For loci with more than one remaining independent *cis* variant, we used inverse-variance weighted MR (MR-IVW). For loci with three or more remaining *cis*-eQTLs, we did sensitivity testing using exposure MR weighted median, MR weighted penalized median, MR weighted mode, MR Egger regression, and MR Egger intercept. MR Steiger was done for every test to assess for reverse causation, wherein the MR results would be better explained by the effects of COVID-19 on the *cis*-eQTL. Results were retained for downstream analysis if they passed these sensitivity tests with a Wald Ratio or IVW Benjamini–Hochberg adjusted p-value, which controls the false-discovery rate by adjusting the p-value by the number of tests, less than or equal to 0.05.

### Colocalization

To investigate whether *cis*-eQTLs shared the same single causal signal with COVID-19 outcomes, we used coloc v5.1.1 (https://chr1swallace.github.io/coloc/). Colocalization helps to guard against bias due to confounding from linkage disequilibrium. Such confounding can occur when the SNPs that influence an exposure (here *cis*-eQTLs) do not causally influence an outcome (here COVID-19 outcomes) but are associated with each other due to linkage disequilibrium. Consistent with Soskic et al., we required at least 50 variants for each colocalization analysis (Soskic et al. 2022). We employed default priors of p_1_ = p_2_ = 10^–4^ and p_12_ = 10^–5^ where p_1_ is the prior probability that only eQTLs had a genetic association in the region, p_2_ is a prior probability that only the HGI summary statistics had a genetic association in the region, and p_12_ is the prior probability that the eQTL data and the HGI summary statistics shared the same genetic associations in the region. A ${p}_{12}\ge 0.8$, or $P{P}_{H4}\ge 0.8$ was considered evidence of colocalization. Sensitivity tests were conducted using coloc’s sensitivity test function for each instance of colocalization to validate the single causal variant assumption and evaluate the robustness of results to different prior settings. Locuszoom plots used to highlight trends were produced using Locuszoom (Pruim et al. 2010).

### Supplementary Information

Below is the link to the electronic supplementary material.

Supplementary file1 (PDF 306 KB)

Supplementary file2 Supplemental Table 1. All lead variants in HGI European-ancestry data, excluding MHC loci. Supplemental Table 2. All genes that were estimated causal and colocalized in our study, with existing evidence linking genes to outcomes. Supplemental Table 3. All MR results using CD4+ T cell expression data. Supplemental Table 4. All MR results using expression data from individuals with symptoms of COVID-19 in BQC19. Supplemental Table 5. All MR results using expression data from all tissues in GTEx. Supplemental Table 6. All colocalization results of putatively causal variants from CD4+ T cell expression data. Supplemental Table 7. All colocalization results of putatively causal variants from individuals with symptoms of COVID-19 in BQC19. Supplemental Table 8. All colocalization results of putatively causal variants from all tissues in GTEx. (XLSX 24172 KB)

## Data Availability

Data from Soskic et al. are available through Zenodo (10.5281/zenodo.6006796). BQC-19 data, including expression data, is available by application: https://www.bqc19.ca/. GTEx release 8 data is available from: https://www.gtexportal.org/home/. COVID-19 HGI summary statistics are available from: https://www.covid19hg.org/.

## Code availability

All analyses were conducted in R v4.0.5 (R Core Team 2021). Code is available at: https://github.com/richardslab/dynamic_QTL_COVID19.
