# Supplementary material for: Colocalization of expression transcripts with COVID-19 outcomes is rare across cell states, cell types and organs
Source: Hum Genet. 2023 Aug 28;142(10):1461–76. doi: 10.1007/s00439-023-02590-w (PMC10511363; doi:10.1007/s00439-023-02590-w)
Supplement: Supplementary file 1 — Supplementary file1 (PDF 306 KB) [file 439_2023_2590_MOESM1_ESM.pdf]

Supplemental Table 2.

Gene	Colocalizing cell/tissue	Existing evidence
ABO	Adrenal gland (Severe) - Hazardous<br>Artery tibial (Severe) – Hazardous<br>Breast Mammary Tissue (Susceptible) – Hazardous<br>Esophagus mucosa (Susceptible) - Hazardous<br>Esophagus muscularis (Severe) - Hazardous<br>Heart Left Ventricle (Severe and Hospitalized) – Hazardous<br>Liver (Hospitalized) - Hazardous<br>Muscle skeletal (Severe) - Hazardous<br>Testis (All) - Hazardous<br>Whole blood COVID+ Symptoms+ (Hospitalized) - **Protective**	MR in non-GTEx and GTEx eQTL and pQTL datasets for lung and whole blood (Baranova et al. 2022; Hernandez Cordero et al. 2021)
ADAM15	T central memory Stim 5d (Susceptible) - Hazardous	MR with colocalization in GTEx did not observe colocalization (Wang et al. 2022)
ATP11A	Artery Aorta (Hospitalized) - Protective	Fine mapping (Kousathanas et al. 2022)
ATP5PO	Lung (Hospitalized) - Hazardous	Associational (Li et al. 2022)
CNPY4	Testis (Severe) - Protective	None
COPS6	Esophagus muscularis (Hospitalized) - Protective	None
DLX3	Lung (Hospitalized) - Hazardous	MR with colocalization in lung (Hernandez Cordero et al. 2021)
DPP9	Skin sun exposed lower leg (All) - Protective	Associational data in blood (Sharif-Zak et al. 2022)
FAM55C	Esophagus muscularis (Susceptible) - Hazardous	None
GBA	Nerve Tibial (Susceptible) - Hazardous<br>Thyroid (Susceptible) - Hazardous	None
GBAP1	Breast Mammary Tissue (Susceptible) - Hazardous<br>Cultured Fibroblasts (Susceptible) - Hazardous<br>Colon Sigmoid (Susceptible) - Hazardous<br>Lung (Susceptible) - Hazardous<br>Nerve Tibial (Susceptible) - Hazardous<br>Small Intestine Terminal Ileum (Susceptible) - Hazardous<br>Stomach (Susceptible) - Hazardous	MR with colocalization for splice isoform (Nakanishi et al. 2022)
HIP1	Breast mammary tissue (Severe) - Hazardous<br>Nerve tibial (Severe) - Hazardous<br>Whole blood COVID- Symptoms- (Severe) - **Protective**	None
IFNAR2	Cells cultured fibroblasts (Severe and hospitalized) - Protective<br>Esophagus mucosa (Susceptible) - Protective<br>Whole blood COVID- Symptoms+ (Hospitalized) - Protective<br>Whole blood COVID+ Symptoms+ (Severe) - Protective	MR with colocalization for GTEx v.7 eQTLs and pQTLs for whole-blood and lung (Baranova et al. 2021; Fricke-Galindo et al. 2022; Krishnamoorthy et al. 2023; Liu et al. 2021; Pairo-Castineira et al. 2021)
IL10RB	Cells Cultured fibroblasts (All) - **Protective**<br>Esophagus Gastroesophageal junction (All) - Hazardous<br>Skeletal muscle (Susceptible) - Hazardous<br>Nerve tibial (All) - Hazardous	MR with colocalization for eQTLs and pQTLs, did not study excitatory neurons (Gaziano et al. 2021)
KCNC3	Tibial nerve (Severe and Hospitalized) – Hazardous	TWAS (Krishnamoorthy et al. 2023)
LINC01301	Breast mammary tissue (Hospitalized) – Protective<br>Skin not sun exposed (Hospitalized) - Protective	TWAS (Pairo-Castineira et al. 2022)
LINC02210	Artery tibial (Severe) – Protective	MR with colocalization (Baranova et al. 2021)
Lnc-INTS12-1	Artery aorta (Severe) – Protective<br>Thyroid (Severe) - Protective	None
Lnc-THBS3-1	Whole blood COVID- Symptoms- (Susceptible) - Protective	None
MAMSTR	Skin Sun Exposed (Hospitalized) – Protective	TWAS (Krishnamoorthy et al. 2023)

MUC1	Nerve tibial (Severe and Hospitalized) - Hazardous	MR from GTEx v.7 against HGI release 6 found signal in blood (Krishnamoorthy et al. 2023)
MUC5B	Lung (Severe) - Protective	MR only for lung eQTLs GTEx release 7 (Liu et al. 2021)
NAPSA	CD4 Naïve Stim 40h (Susceptible) - Hazardous<br>Esophageal mucosa (Severe and Hospitalized) - **Protective**<br>T effector memory Stim 40h (All) - Hazardous<br>T naïve (Hospitalized and Susceptible) - Hazardous<br>Testis (Hospitalized) - **Protective**	MR with colocalization for eQTLs, did not study CD4+ T cells (Degenhardt et al. 2022)
NAPSB	Artery Coronary (Susceptible) - Protective	None
NTN5	Colon sigmoid (Hospitalized) - Protective<br>Esophageal Gastroesophageal junction (Hospitalized) - Protective<br>Esophagus mucosa (Severe and Hospitalized) - Protective	None
OAS1	Adipose subcutaneous (Hospitalized) - Protective<br>Artery tibial (Susceptible) - Protective<br>Esophagus mucosa (Hospitalized) - **Hazardous**<br>Nerve tibial (Susceptible) - **Hazardous**<br>Skin not sun exposed (Severe and Susceptible) - Protective<br>Skin sun exposed (All) - Protective	MR with colocalization of eQTL and pQTL (Zhou et al. 2021)
PDCL3P4	Brain Caudate Basal Ganglia (Susceptible)	Colocalization (D'Antonio et al. 2021)
RAB2A	Adipose subcutaneous (Hospitalized) - Hazardous<br>Artery aorta (Hospitalized) - Hazardous<br>Artery tibial (Hospitalized) - Hazardous<br>CD4 Memory Stim 40h (Hospitalized) - Hazardous<br>CD4 Naïve Stim 16h (Hospitalized) - Hazardous<br>CD4 Naïve Stim 40h (Hospitalized) - Hazardous<br>Esophagus mucosa (Hospitalized) - Hazardous<br>Esophagus muscularis (Hospitalized) - Hazardous<br>Liver (Hospitalized) - Hazardous<br>Lung (Hospitalized) - Hazardous<br>Skin not sun exposed (Hospitalized) - Hazardous<br>T naïve Stim 40h (Hospitalized) - Hazardous	MR and colocalization for pQTLs, but not for eQTLs (Pietzner et al. 2022)
RALGDS	T effect memory Stim 16h (Severe and Susceptible) - Protective	None
RASIP1	Esophageal Gastroesophageal junction (Severe and Hospitalized) - Protective<br>Esophagus muscularis (Severe) - Protective	None
SLC22A31	Heart left ventricle (Severe and Hospitalized) – Hazardous	PheWAS linked (Covid-19 Host Genetics Initiative 2022)
SLC6A20	Breast mammary tissue (Severe and Hospitalized) – Hazardous	GWAS (Severe Covid et al. 2020)
WNT3	Adrenal Gland (Hospitalized) - Hazardous<br>Artery Aorta (Severe and Hospitalized) - **Protective**<br>Colon Transverse (Severe) - Hazardous<br>Nerve Tibial (Severe) - Hazardous<br>Thyroid (Severe and Hospitalized) - Hazardous	Fine mapped (Wu et al. 2021)

References

Baranova A, Cao H, Chen J, Zhang F (2022) Causal Association and Shared Genetics Between Asthma and COVID-19. Front Immunol 13: 705379. doi: 10.3389/fimmu.2022.705379

Baranova A, Cao H, Zhang F (2021) Unraveling Risk Genes of COVID-19 by Multi-Omics Integrative Analyses. Front Med (Lausanne) 8: 738687. doi: 10.3389/fmed.2021.738687

Covid-19 Host Genetics Initiative (2022) A first update on mapping the human genetic architecture of COVID-19. Nature 608: E1-E10. doi: 10.1038/s41586-022-04826-7

D'Antonio M, Nguyen JP, Arthur TD, Matsui H, Initiative C-HG, D'Antonio-Chronowska A, Frazer KA (2021) SARS-CoV-2 susceptibility and COVID-19 disease severity are associated with genetic variants affecting gene expression in a variety of tissues. Cell Rep 37: 110020. doi: 10.1016/j.celrep.2021.110020

Degenhardt F, Ellinghaus D, Juzenas S, Lerga-Jaso J, Wendorff M, Maya-Miles D, Uellendahl-Werth F, ElAbd H, Ruhlemann MC, Arora J, Ozer O, Lenning OB, Myhre R, Vadla MS, Wacker EM, Wienbrandt L, Blandino Ortiz A, de Salazar A, Garrido Chercoles A, Palom A, Ruiz A, Garcia-Fernandez AE, Blanco-Grau A, Mantovani A, Zanella A, Holten AR, Mayer A, Bandera A, Cherubini A, Protti A, Aghemo A, Gerussi A, Ramirez A, Braun A, Nebel A, Barreira A, Lleo A, Teles A, Kildal AB, Biondi A, Caballero-Garralda A, Ganna A, Gori A, Gluck A, Lind A, Tanck A, Hinney A, Carreras Nolla A, Fracanzani AL, Peschuck A, Cavallero A, Dyrhol-Riise AM, Ruello A, Julia A, Muscatello A, Pesenti A, Voza A, Rando-Segura A, Solier A, Schmidt A, Cortes B, Mateos B, Nafria-Jimenez B, Schaefer B, Jensen B, Bellinghausen C, Maj C, Ferrando C, de la Horra C, Quereda C, Skurk C, Thibeault C, Scollo C, Herr C, Spinner CD, Gassner C, Lange C, Hu C, Paccapelo C, Lehmann C, Angelini C, Cappadona C, Azuure C, Covicat study group AS, Bianco C, Cea C, Sancho C, Hoff DAL, Galimberti D, Prati D, Haschka D, Jimenez D, Pestana D, Toapanta D, Muniz-Diaz E, Azzolini E, Sandoval E, Binatti E, Scarpini E, Helbig ET, et al. (2022) Detailed stratified GWAS analysis for severe COVID-19 in four European populations. Hum Mol Genet 31: 3945-3966. doi: 10.1093/hmg/ddac158

Fricke-Galindo I, Martinez-Morales A, Chavez-Galan L, Ocana-Guzman R, Buendia-Roldan I, Perez-Rubio G, Hernandez-Zenteno RJ, Veronica-Aguilar A, Alarcon-Dionet A, Aguilar-Duran H, Gutierrez-Perez IA, Zaragoza-Garcia O, Alanis-Ponce J, Camarena A, Bautista-Becerril B, Nava-Quiroz KJ, Mejia M, Guzman-Guzman IP, Falfan-Valencia R (2022) IFNAR2 relevance in the clinical outcome of individuals with severe COVID-19. Front Immunol 13: 949413. doi: 10.3389/fimmu.2022.949413

Gaziano L, Giambartolomei C, Pereira AC, Gaulton A, Posner DC, Swanson SA, Ho YL, Iyengar SK, Kosik NM, Vujkovic M, Gagnon DR, Bento AP, Barrio-Hernandez I, Ronnblom L, Hagberg N, Lundtoft C, Langenberg C, Pietzner M, Valentine D, Gustincich S, Tartaglia GG, Allara E, Surendran P, Burgess S, Zhao JH, Peters JE, Prins BP, Angelantonio ED, Devineni P, Shi Y, Lynch KE, DuVall SL, Garcon H, Thomann LO, Zhou JJ, Gorman BR, Huffman JE, O'Donnell CJ, Tsao PS, Beckham JC, Pyarajan S, Muralidhar S, Huang GD, Ramoni R, Beltrao P, Danesh J, Hung AM, Chang KM, Sun YV, Joseph J, Leach AR, Edwards TL, Cho K, Gaziano JM, Butterworth AS, Casas JP, Initiative VAMVPC-S (2021) Actionable druggable genome-wide Mendelian randomization identifies repurposing opportunities for COVID-19. Nat Med 27: 668-676. doi: 10.1038/s41591-021-01310-z

Hernandez Cordero AI, Li X, Milne S, Yang CX, Bosse Y, Joubert P, Timens W, van den Berge M, Nickle D, Hao K, Sin DD (2021) Multi-omics highlights ABO plasma protein as a causal risk factor for COVID-19. Hum Genet 140: 969-979. doi: 10.1007/s00439-021-02264-5

Kousathanas A, Pairo-Castineira E, Rawlik K, Stuckey A, Odhams CA, Walker S, Russell CD, Malinauskas T, Wu Y, Millar J, Shen X, Elliott KS, Griffiths F, Oosthuyzen W, Morrice K, Keating S, Wang B, Rhodes D, Klaric L, Zechner M, Parkinson N, Siddiq A, Goddard P, Donovan S, Maslove D, Nichol A, Semple MG, Zainy T, Maleady-Crowe F, Todd L, Salehi S, Knight J, Elgar G, Chan G, Arumugam P, Patch C, Rendon A, Bentley D, Kingsley C, Kosmicki JA, Horowitz JE, Baras A, Abecasis GR, Ferreira MAR, Justice A, Mirshahi T, Oetjens M, Rader DJ, Ritchie MD, Verma A, Fowler TA, Shankar-Hari M, Summers C, Hinds C, Horby P, Ling L, McAuley D, Montgomery H, Openshaw PJM, Elliott P, Walsh T, Tenesa A, Gen Oi, andMe i, Initiative C-HG, Fawkes A, Murphy L, Rowan K, Ponting CP, Vitart V, Wilson JF, Yang J, Bretherick AD, Scott RH, Hendry SC, Moutsianas L, Law A, Caulfield MJ, Baillie JK (2022) Whole-genome sequencing reveals host factors underlying critical COVID-19. Nature 607: 97-103. doi: 10.1038/s41586-022-04576-6

Krishnamoorthy S, Li GH, Cheung CL (2023) Transcriptome-wide summary data-based Mendelian randomization analysis reveals 38 novel genes associated with severe COVID-19. J Med Virol 95: e28162. doi: 10.1002/jmv.28162

Li P, Ke Y, Shen W, Shi S, Wang Y, Lin K, Guo X, Wang C, Zhang Y, Zhao Z (2022) Targeted screening of genetic associations with COVID-19 susceptibility and severity. Front Genet 13: 1073880. doi: 10.3389/fgene.2022.1073880

Liu D, Yang J, Feng B, Lu W, Zhao C, Li L (2021) Mendelian randomization analysis identified genes pleiotropically associated with the risk and prognosis of COVID-19. J Infect 82: 126-132. doi: 10.1016/j.jinf.2020.11.031

Nakanishi T, Farjoun Y, Willett J, Allen RJ, Guillen-Guio B, Zhou S, Richards JB (2022) Alternative splicing in the lung influences COVID-19 severity and respiratory diseases. medRxiv. doi: 10.1101/2022.10.18.22281202

Pairo-Castineira E, Clohisey S, Klaric L, Bretherick AD, Rawlik K, Pasko D, Walker S, Parkinson N, Fourman MH, Russell CD, Furniss J, Richmond A, Gountouna E, Wrobel N, Harrison D, Wang B, Wu Y, Meynert A, Griffiths F, Oosthuyzen W, Kousathanas A, Moutsianas L, Yang Z, Zhai R, Zheng C, Grimes G, Beale R, Millar J, Shih B, Keating S, Zechner M, Haley C, Porteous DJ, Hayward C, Yang J, Knight J, Summers C, Shankar-Hari M, Klenerman P, Turtle L, Ho A, Moore SC, Hinds C, Horby P, Nichol A, Maslove D, Ling L, McAuley D, Montgomery H, Walsh T, Pereira AC, Renieri A, Gen OI, Investigators IC, Initiative C-HG, andMe I, Investigators B, Gen CI, Shen X, Ponting CP, Fawkes A, Tenesa A, Caulfield M, Scott R, Rowan K, Murphy L, Openshaw PJM, Semple MG, Law A, Vitart V, Wilson JF, Baillie JK (2021) Genetic mechanisms of critical illness in COVID-19. Nature 591: 92-98. doi: 10.1038/s41586-020-03065-y

Pairo-Castineira E, Rawlik K, Klaric L, Kousathanas A, Richmond A, Millar J, Russell CD, Malinauskas T, Thwaites R, Stuckey A, Odhams CA, Walker S, Griffiths F, Oosthuyzen W, Morrice K, Keating S, Nichol A, Semple MG, Knight J, Shankar-Hari M, Summers C, Hinds C, Horby P, Ling L, McAuley D, Montgomery H, Openshaw PJM, Walsh T, Tenesa A, Scott RH, Caulfield MJ, Moutsianas L, Ponting CP, Wilson JF, Vitart V, Pereira AC, Luchessi A, Parra E, Cruz-Guerrero R, Carracedo A, Fawkes A, Murphy L, Rowan K, Law A, Hendry SC, Baillie JK (2022) GWAS and meta-analysis identifies multiple new genetic mechanisms underlying severe Covid-19. medRxiv. doi: 10.1101/2022.03.07.22271833

Pietzner M, Chua RL, Wheeler E, Jechow K, Willett JDS, Radbruch H, Trump S, Heidecker B, Zeberg H, Heppner FL, Eils R, Mall MA, Richards JB, Sander LE, Lehmann I, Lukassen S, Wareham NJ, Conrad C, Langenberg C (2022) ELF5 is a potential respiratory epithelial cell-specific risk gene for severe COVID-19. Nat Commun 13: 4484. doi: 10.1038/s41467-022-31999-6

Severe Covid GG, Ellinghaus D, Degenhardt F, Bujanda L, Buti M, Albillos A, Invernizzi P, Fernandez J, Prati D, Baselli G, Asselta R, Grimsrud MM, Milani C, Aziz F, Kassens J, May S, Wendorff M, Wienbrandt L, Uellendahl-Werth F, Zheng T, Yi X, de Pablo R, Chercoles AG, Palom A, Garcia-Fernandez AE, Rodriguez-Frias F, Zanella A, Bandera A, Protti A, Aghemo A, Lleo A, Biondi A, Caballero-Garralda A, Gori A, Tanck A, Carreras Nolla A, Latiano A, Fracanzani AL, Peschuck A, Julia A, Pesenti A, Voza A, Jimenez D, Mateos B, Nafria Jimenez B, Quereda C, Paccapelo C, Gassner C, Angelini C, Cea C, Solier A, Pestana D, Muniz-Diaz E, Sandoval E, Paraboschi EM, Navas E, Garcia Sanchez F, Ceriotti F, Martinelli-Boneschi F, Peyvandi F, Blasi F, Tellez L, Blanco-Grau A, Hemmrich-Stanisak G, Grasselli G, Costantino G, Cardamone G, Foti G, Aneli S, Kurihara H, ElAbd H, My I, Galvan-Femenia I, Martin J, Erdmann J, Ferrusquia-Acosta J, Garcia-Etxebarria K, Izquierdo-Sanchez L, Bettini LR, Sumoy L, Terranova L, Moreira L, Santoro L, Scudeller L, Mesonero F, Roade L, Ruhlemann MC, Schaefer M, Carrabba M, Riveiro-Barciela M, Figuera Basso ME, Valsecchi MG, Hernandez-Tejero M, Acosta-Herrera M, D'Angio M, Baldini M, Cazzaniga M, Schulzky M, Cecconi M, Wittig M, et al. (2020) Genomewide Association Study of Severe Covid-19 with Respiratory Failure. N Engl J Med 383: 1522-1534. doi: 10.1056/NEJMoa2020283

Sharif-Zak M, Abbasi-Jorjandi M, Asadikaram G, Ghoreshi ZA, Rezazadeh-Jabalbarzi M, Afsharipur A, Rashidinejad H, Khajepour F, Jafarzadeh A, Arefinia N, Kheyrkhah A, Abolhassani M (2022) CCR2 and DPP9 expression in the peripheral blood of COVID-19 patients: Influences of the disease severity and gender. Immunobiology 227: 152184. doi: 10.1016/j.imbio.2022.152184

Wang Y, Guga S, Wu K, Khaw Z, Tzoumkas K, Tombleson P, Comeau ME, Langefeld CD, Cunninghame Graham DS, Morris DL, Vyse TJ (2022) COVID-19 and systemic lupus erythematosus genetics: A balance between autoimmune disease risk and protection against infection. PLoS Genet 18: e1010253. doi: 10.1371/journal.pgen.1010253

Wu L, Zhu J, Liu D, Sun Y, Wu C (2021) An integrative multiomics analysis identifies putative causal genes for COVID-19 severity. Genet Med 23: 2076-2086. doi: 10.1038/s41436-021-01243-5

Zhou S, Butler-Laporte G, Nakanishi T, Morrison DR, Afilalo J, Afilalo M, Laurent L, Pietzner M, Kerrison N, Zhao K, Brunet-Ratnasingham E, Henry D, Kimchi N, Afrasiabi Z, Rezk N, Bouab M, Petitjean L, Guzman C, Xue X, Tselios C, Vulesevic B, Adeleye O, Abdullah T, Almamlouk N, Chen Y, Chasse M, Durand M, Paterson C, Normark J, Frithiof R, Lipcsey M, Hultstrom M, Greenwood CMT, Zeberg H, Langenberg C, Thysell E, Pollak M, Mooser V, Forgetta V, Kaufmann DE, Richards JB (2021) A Neanderthal OAS1 isoform protects individuals of European ancestry against COVID-19 susceptibility and severity. Nat Med 27: 659-667. doi: 10.1038/s41591-021-01281-1
